# Multiple Subtypes of Alzheimer’s Disease Base on Brain Atrophy Pattern

**DOI:** 10.3390/brainsci11020278

**Published:** 2021-02-23

**Authors:** Baiwen Zhang, Lan Lin, Shuicai Wu, Zakarea H. M. A. Al-Masqari

**Affiliations:** 1 Department of Biomedical Engineering, Faculty of Environment and Life Sciences, Beijing University of Technology, Beijing 100124, China; zhangby@emails.bjut.edu.cn (B.Z.); wushuicai@bjut.edu.cn (S.W.); zakarea@emails.bjut.edu.cn (Z.H.M.A.A.-M.); 2 Intelligent Physiological Measurement and Clinical Translation, Beijing International Base for Scientific and Technological Cooperation, Beijing University of Technology, Beijing 100124, China

**Keywords:** Alzheimer’s disease, atrophy subtypes, cortical thickness, mixture of experts, structural magnetic resonance imaging, neuropsychology, neuropathology

## Abstract

Alzheimer’s disease (AD) is a disease of a heterogeneous nature, which can be disentangled by exploring the characteristics of each AD subtype in the brain structure, neuropathology, and cognition. In this study, a total of 192 AD and 228 cognitively normal (CN) subjects were obtained from the Alzheimer’s disease Neuroimaging Initiative database. Based on the cortical thickness patterns, the mixture of experts method (MOE) was applied to the implicit model spectrum of transforms lined with each AD subtype, then their neuropsychological and neuropathological characteristics were analyzed. Furthermore, the piecewise linear classifiers composed of each AD subtype and CN were resolved, and each subtype was comprehensively explained. The following four distinct AD subtypes were discovered: bilateral parietal, frontal, and temporal atrophy AD subtype (occipital sparing AD subtype (OSAD), 29.2%), left temporal dominant atrophy AD subtype (LTAD, 22.4%), minimal atrophy AD subtype (MAD, 16.1%), and diffuse atrophy AD subtype (DAD, 32.3%). These four subtypes display their own characteristics in atrophy pattern, cognition, and neuropathology. Compared with the previous studies, our study found that some AD subjects showed obvious asymmetrical atrophy in left lateral temporal-parietal cortex, OSAD presented the worst cerebrospinal fluid levels, and MAD had the highest proportions of APOE ε4 and APOE ε2. The subtype characteristics were further revealed from the aspect of the model, making it easier for clinicians to understand. The results offer an effective support for individual diagnosis and prognosis.

## 1. Introduction

Alzheimer’s disease (AD) is a heterogeneous disease. Memory impairment is a common characteristic among AD patients; some, however, suffer from other obvious cognitive deficits, such as executive function impairment or language impairment [1]. A post-mortem study pointed out that the tau protein tangles showed different development rules among about 25% of AD subjects, and these samples were classified into two subtypes: hippocampal-sparing AD (HSAD) and limbic-predominant AD (LPAD) [2]. In an imaging study, the atrophy features of gray matter were extracted via structural magnetic resonance imaging (sMRI), the subtypes similar to those by Murray et al. and obtained the subtypes presented different neuropathological and neuropsychological characteristics [3].

The cortical atrophy features acquired from sMRI have been applied to the research on early-stage AD subtypes [4]. In some studies, the subtypes have been divided by calculating the ratio of cortex to regions of interest (ROIs), which is highly correlated with AD [5,6,7]. The subtypes have also been identified via visual rating scales [8,9]. However, these methods, which belong to priori hypothesis, need to preset the subtypes and their corresponding ROIs. Moreover, the AD subtypes have been defined through the clustering method [10,11,12], where the ROIs presenting is not needed, and new atrophy subtypes may be discovered. For instance, the mild atrophy AD subtype (MAD) has been found in a study [10], and the HSAD has been further refined into some regions in the parietal lobe. Clustering emphasizes on the similarity between individuals, and the results depend on the data contribution to the model, so nuisance variables unrelated to AD itself, such as age, gender, or other neurological diseases, may influence the clustering result [13]. By establishing multiple different disease conversion models, the semi-supervised algorithm can capture the pathological differences between cognitively normal (CN) and AD and distinguish heterogeneous disease effects during the occurrence and development of AD. CHIMERA is an approach that adopts the probabilistic clustering to construct multiple regularized CN-to-AD conversion models, so as to simulate the pathological process of AD and differentiate its subtypes [14]. In a recent study, multiple convex polytopes were constructed between CN and AD through the linear support vector machine (SVM), and the characteristics of AD subtypes were reflected by the information of each polytope [15]. In this model, a linear SVM corresponding to each subtype was formed, and, in comparison with the nonlinear model (e.g., CHIMERA), the interpretability of each AD subtype was enhanced. However, this framework divided the subtypes only by depending on the distance from subjects to hyperplanes, lacking the clustering of AD subjects.

The mixture of experts (MOE) that integrates the fuzzy clustering (mixture) with piece-wise linear SVM (expert) [16]. In this model, the clustering method and SVM are alternately optimized, namely, the affected groups, while being clustered, established multiple variable patterns with the reference group. This model has been applied to explore the heterogeneity of brain aging [17]. In this study, the MOE was used to investigate AD subtypes. It was assumed that multiple variable trajectories existed from CN to AD, and multilinear maps were then established between the AD subtypes and CN, thus providing a clue for exploring the AD subtypes.

## 2. Materials and Methods

### 2.1. Participants and MRI Processing

The data used in this study were acquired from the Alzheimer’s disease Neuroimaging Initiative (ADNI) database (www.adni.loni.usc.edu) (accessed on 23 February 2021) [18]. ADNI, which was initially set up in 2004, includes MRIs, neuropsychological and neuropathological data. It had recruited about 800 subjects, ages 55–90 years, in the first phase (ADNI-1), including the CN individuals, patients with AD or mild cognitive impairment (MCI). The inclusion or exclusion criteria have been explicitly described by Petersen et al. [19].

T1-weighted structural MR scans (voxel size 1.1 × 1.1 × 1.2 mm^3^) from 192 AD and 188 CN participants of the ADNI-1 baseline sample were considered in this study [20]. The cortical reconstruction and the volume segmentation were completed by Freesurfer version 4.3 (http://freesurfer.net/) (accessed on 23 February 2021) [21]. The cortical and subcortical information of gray matter thickness based on the Desikan–Killiany atlas were provided by the ADNI website, which could be used for the subsequent analyses.

### 2.2. Neuropsychological Assessment and Neuropathological Data Collection

The neuropsychological data used in this study could be divided into global cognitive scales, Functional Activities Questionnaire (FAQ), and ADNI-composite scores. Among them, the first mainly includes Mini-Mental State Exam (MMSE), Clinical Dementia Rating Scale-Sum of Boxes (CDR-SB), and AD Assessment Scale-Cognitive Subscale (ADAS-Cog), and the last includes four sub-domains: memory, executive function, language, and visuospatial. Gibbons et al. derived the composite scores for memory (ADNI-MEM) and executive function (ADNI-EF) according to the ADNI neuropsychological battery using item response theory (IRT) methods in 2012 [22,23,24], and Choi et al. designed the composite scores for language (ADNI-LAN) and visuospatial abilities (ADNI-VS) using similar methods in 2020 [22]. Their basic items are shown in Figure 3.

All subjects contained the apolipoprotein E (APOE) information with two alleles, and the cerebrospinal fluid (CSF) data was acquired from about half of the subjects. Of 228 CN participants, 111 had CSF data available. Of 192 AD participants, 98 had their CSF beta-amyloid 1–42 (Aβ1–42) and phosphorylated tau (p-tau) data available, and CSF total tau (t-tau) data was discovered in 96 ones [22,25,26].

### 2.3. Definition of AD Subtype Using MOE

Sixty-eight regional cortical thickness features were obtained from the Desikan–Killiany Atlas for defining the AD subtypes in each subject. In order to minimize the effects of individual differences, the age, sex, years of education and intracranial volume (ICV) were termed as extraneous variables. For each feature, the regression coefficient of CN ($\beta_{\mathrm{CN}}$) was estimated by the generalized linear model (GLM):

(1) $$\mathrm{thickness}\text{\_}{\mathrm{value}}_{\mathrm{CN}}=\beta_{\mathrm{CN}}\times \left(1+{\mathrm{age}}_{\mathrm{CN}}+{\mathrm{sex}}_{\mathrm{CN}}+{\mathrm{edu}}_{\mathrm{CN}}+{\mathrm{ICV}}_{\mathrm{CN}}\right)+\epsilon$$

Then, the effects of age, sex, years of education and ICV were regressed out of all subjects (AD and CN):

(2) $${\mathrm{residual}}_{\mathrm{ALL}}=\mathrm{thickness}\text{\_}{\mathrm{value}}_{\mathrm{ALL}}-\beta_{\mathrm{CN}}\times \left(1+{\mathrm{age}}_{\mathrm{ALL}}+{\mathrm{sex}}_{\mathrm{ALL}}+{\mathrm{edu}}_{\mathrm{CN}}+{\mathrm{ICV}}_{\mathrm{ALL}}\right)$$

**Figure 1:**
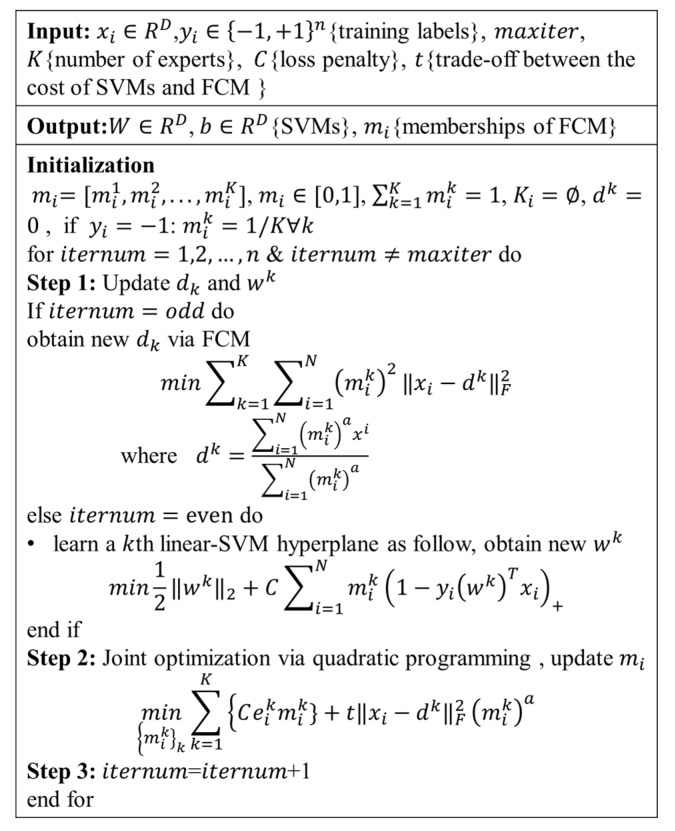
The algorithm procedure of the mixture of experts (MOE) method.

The algorithm flowchart of MOE is shown in Figure 1. The CN subjects served as the reference group (y=−1), and the AD subject as the affected group (y=1). The cross-validation was used (f=10), and the number of experts depended on several validation indexes as follows: SVM accuracy, maximum pair-wise inner-product, and cluster separation index (BPC) [27]. C and t were evaluated via grid-based the research, and they range within $\left\{2^{-3},2^{-2},\ldots 2^{10}\right\}$.

### 2.4. Evaluation of Multiple Piece-Wise Linear SVM

After the AD subtypes were defined, the cross-validation (f=5) was performed for each SVM classifier of AD subtype and NC in the MOE. The performance of the methods was evaluated on SVMs, using accuracy (ACC), sensitivity (SEN), specificity (SPE):

(3) $$\mathrm{ACC}=\frac{\mathrm{TP}+\mathrm{TN}}{\mathrm{TP}+\mathrm{PN}+\mathrm{FP}+\mathrm{FN}}$$

(4) $$\mathrm{SEN}=\frac{\mathrm{TP}}{\mathrm{TP}+\mathrm{FN}}$$

(5) $$\mathrm{SPE}=\frac{\mathrm{TN}}{\mathrm{TN}+\mathrm{FP}}$$

### 2.5. Statistical Analyses

Group analyses were performed using SPSS, version 19.0, Armonk, NY, USA. The demographic variables, cognitive test scores, APOE genotype (APOE ε2 and APOE ε4), and CSF biomarker levels were compared among the AD subtypes. The quantitative variables were compared via the analysis of variance (ANOVA), and pairwise comparison via the Dunnett’s test. On this basis, the significant difference between two AD subtype groups were reported. The differences among the qualitative variables were calculated using the chi-square tests, and the abnormal proportions were figured out after the missing data were excluded. 

Next, the longitudinal analysis was conducted for AD subjects. In each subtype, the cortical thickness data of 68 ROIs were used to calculate the annual rate of change. The cortical thickness data were input into the hyperplanes and parameters of the MOE model, which were generated in the definition of AD subtypes, and then the time-varying subtype attribution of each subject was calculated. The longitudinal scores of each subtype on the global cognition scales, FAQ and the ADNI-composite scores in 4 sub-domains were also calculated. In all longitudinal analyses, the data used were sampled at 12 months or 24 months.

## 3. Results

### 3.1. AD Subtypes Identified by MOE

**Figure 2:**
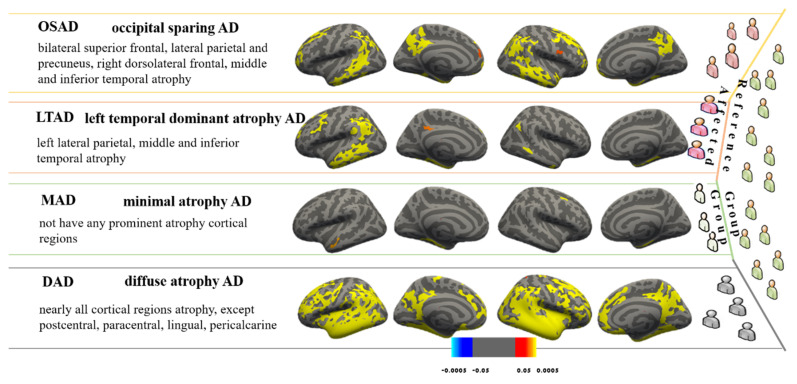
These subtypes were identified from MOE compared with controls. Effect size maps are thresholded at false discovery rate (FDR) adjusted *p*-value of 0.0005.

The AD subjects were divided into 4 subtypes using the MOE method. The 4 subgroups from the CN group manifested a reasonable classification and separation, with the average accuracy of 83.1 ± 4.8%, the BPC of 0.63 ± 0.02, and the maximum pair-wise inner product equal for 0.29 ± 0.07. Each subtype was named after its statistical characteristic—cortical thickness atrophy pattern—found in comparison with the CN cohort (Figure 2). The subjects were divided into the followings: (1) Occipital sparing AD subtype (OSAD, *n* = 56, 29.2%), a group showed prominent bilateral parietal and frontal atrophy, mainly including bilateral lateral parietal lobes, precuneus, bilateral superior frontal, middle and inferior temporal. (2) Left temporal dominant atrophy AD subtype (LTAD, *n* = 43, 22.4%), a group displayed the prominent atrophy in the left lateral parietal, middle, and inferior temporal. (3) Minimal atrophy AD subtype (MAD, *n* = 31, 16.1%), a group presented the least extent and amount of atrophy among 4 groups, only suffering from sporadic atrophy in the inferior temporal of the whole cortical cortex. (4) Diffuse atrophy AD subtype (DAD, *n* = 62, 32.3%), a group with atrophy in nearly all cortical regions except for postcentral, paracentral lingual, and pericalcarine areas. 

### 3.2. Demographic and Cognitive Characteristics among Four AD Subtypes

**Figure 3:**
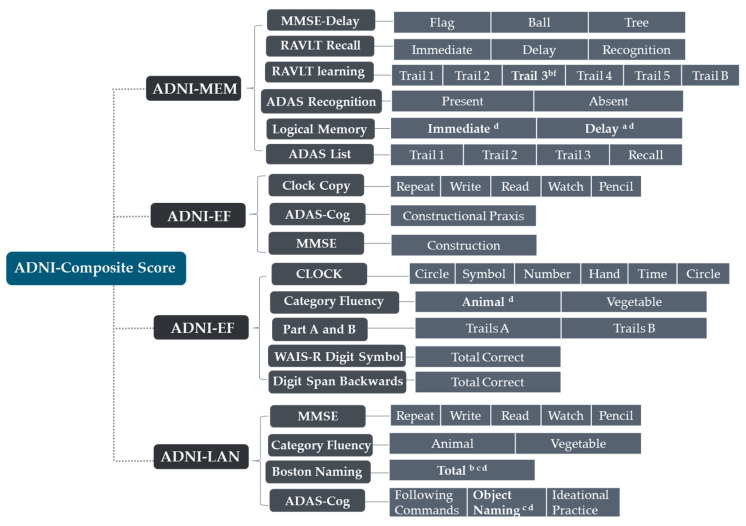
The items of Alzheimer’s Disease Neuroimaging Initiative (ADNI)-composite scores. ^a:^ significant differences (*p* < 0.05) between OSAD and MAD; ^b:^ significant differences (*p* < 0.05) between OSAD and DAD; ^c:^ significant differences (*p* < 0.05) between LTAD and DAD; ^d:^ significant differences (*p* < 0.05) between MAD and DAD.

The demographic information of the four AD subtypes and the CN group were compared (Table 1). The LTAD subjects who were averagely oldest, with relatively high-level education, while the situation was the contrary among the MAD subjects. However, no statistical differences were found among the four subtypes in age, years of education, handedness or vital characteristics. There was no significant difference in the age of onset among the subtypes, either. The LTAD subjects had the shortest disease duration, in which aspect they were significantly different from DAD and OSAD. The females accounted for the minimum proportion in LTAD patients who were significantly different from OSAD and DAD with respect to this. The DAD subtype showed the most obvious cognitive impairment, and it had significant differences from the other three subtypes in ADAS-Cog 11, ADAS-Cog 13, ADNI-MEM, and ADNI-LAN (Figure 3). Except for DAD, the average scores of MMSE of three subtypes were nearly equal. The MAD had higher cognitive scores than other three subtypes, and its differences from other groups were also significant in FAQ and ADNI-MEM. 

### 3.3. Longitudinal Changes among Four Subtypes of AD

**Figure 4:**
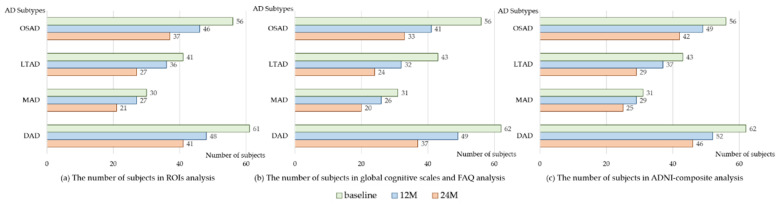
The number of subjects in longitudinal analysis. 12M, at 12-month follow-up; 24M, at 24-month follow-up; OSAD, occipital sparing Alzheimer’s Disease (AD) subtype; LTAD, left temporal dominant atrophy AD subtype; MAD, minimal atrophy AD subtype; DAD, diffuse atrophy AD subtype.

**Figure 5:**
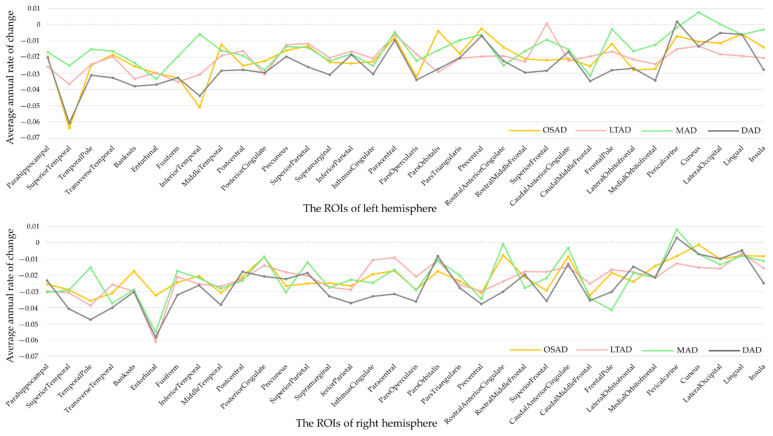
Rate of changes in regions of interest. ROIs, regions of interest.

**Figure 6:**
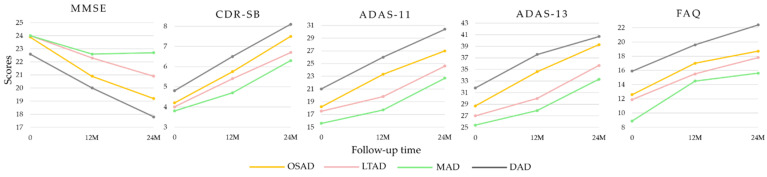
Longitudinal cognitive changes in global cognitive scales and FAQ. 0, baseline.

**Figure 7:**
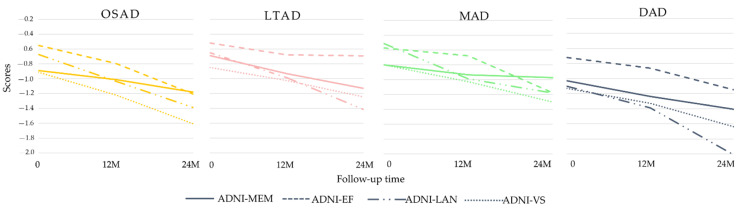
Longitudinal cognitive changes in ADNI-composite scores.

**Figure 8:**
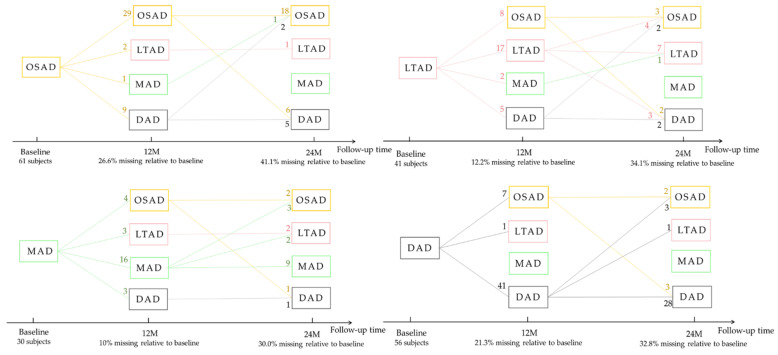
Subtype changes with the progression of disease. The number on each line represents the number of subjects who are transformed from the current subtype into another subtype or remained itself.

Some AD subjects were not involved in the longitudinal data collection. The number of subjects was counted in the longitudinal analysis (Figure 4). The average rates of change in the cortical thickness of ROIs among the different subtypes were summarized (Figure 5). The cortical thickness of MAD changed little in most of ROIs, while that of DAD was on the contrary. LTAD showed obvious changes in bilateral occipital and temporal lobes. In global cognitive scales and FAQ, DAD and OSAD showed a faster progression from the baseline to 24M. Although the MMSE scores were almost equal to the baseline of OSAD, MAD, and LTAD, MAD decreased less while OSAD decreased more over time (Figure 6). In the ADNI-composite scores, DAD showed an overall decline in all cognitive domains, while MAD was the opposite. Compared with MAD and OSAD, the memory ability and language ability of LTAD declined faster (Figure 7). The four subtypes would be changed, to some extent, with the progression of disease. Among them, LTAD was converted into other subtypes relatively more frequently, but the majority of AD subjects maintained the subtype type of their respective baseline (Figure 8).

### 3.4. Neuropathological Characteristics among Four AD Subtypes

The proportions of APOE ε4 alleles carried in all subgroups were higher than 60%. The proportions of APOE ε4 and APOE ε2 carried in MAD were the maximum, where the APOE ε2 in MAD was significantly different from those in the other groups. The DAD group presented the highest proportion of APOE ε4 homozygote. In the quantitative analysis of Aβ_1–42_, the concentration of Aβ_1–42_ in MAD was significantly higher than those in OSAD and DAD. The average Aβ_1–42_ in OSAD was the minimum, and their abnormality ratio was the highest among the four subtypes. The normal tau ratio in MAD was highest in all four subtypes, the abnormality ratios of t-tau and p-tau in OSAD seemed to be the highest in AD subtype, and they were significantly different from those in MAD through the qualitative analysis (Table 2). 

### 3.5. The Classification Performance of Multiple Piece-Wise Linear SVMs

**Figure 9:**
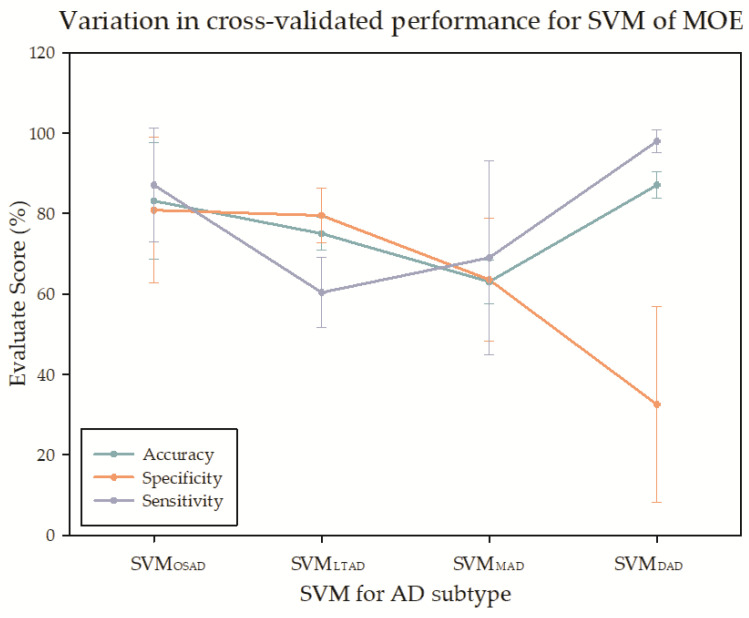
The cross-validation for the support vector machine (SVM) of MOE.

In the multiple linear SVMs constituted by CN and AD subtypes, the classifier between DAD and CN presented the best classification accuracy and sensitivity. Still, the specificity was the lowest among the four subgroups. The accuracy was the lowest in MAD, in which the sensitivity was only higher than that in LTAD, and the specificity was only higher than that in DAD (Figure 9).

## 4. Discussion

In this study, the cortical thickness patterns from sMRI were applied to establish multiple AD subtypes and CN linear classifiers, and meanwhile, the clustering was implemented within AD, and the potential neuropathologic CN to AD subtypes change processes were simulated. It was found that the structural features were evidently different among the four AD subtypes, and so were the corresponding multiple cognitive tests and pathological markers. Therefore, the brain changes of AD subtypes will experience heterogeneous structural, cognitive, and pathological processes during the development of CN to AD.

From the atrophy patterns of each subtype, the OSAD showed obvious atrophy of the bilateral frontal lobes, lower parietal lobe, and precuneus, which was similar to the HSAD in the previous studies. The LTAD showed evident atrophy in left lateral temporal-parietal lobe, but the atrophy regions in right and medial left hemisphere were small. No obvious cortical thickness atrophy appeared in MAD. Just like the typical AD in previous studies, the DAD presented the diffuse atrophy in the whole cortex. To date, the proportion of typical AD, only one study (named: diffuse AD) exceeded 75% in Murray et al.’s study [2,28], but it was always lower than 75% under most circumstances, and that obtained by Kate et al. did not even reach 20% [10]. The main reason is that Murray et al., obtained the proportion based on the autopsy results of the subjects that were in the late stage of AD and died of AD, while the AD subjects in databases (e.g., ADNI) were in the early stage of late-onset AD.

The LTAD was manifested by obvious asymmetrical atrophy in left lateral hemisphere. In previous studies, the subtypes were named directly by the regions of atrophy like the left hemisphere, but through the hierarchical clustering in a study, the left-right asymmetrical cerebral atrophy was discovered in the HSAD [12], while an obvious atrophy in left hemisphere was also presented in the subtype of Lateral Temporal-Language [29]. According to another, the glucose metabolic levels in the left hippocampus and frontal lobe were obviously lower than those on the right side [11]. This asymmetry has also been mentioned in other studies regarding AD. It has been confirmed that the left parietal atrophy takes place in over 80% of the early-stage AD [30,31]. Evidence also showed that the tau deposition and gray matter loss in left temporal of some patients were obviously higher than those at the right side, accompanied by the prominent language disorder [22,30,32]. In the subitem calculations of LTAD in this study, it was found that the vegetables in Boston Total and Category Fluency of LTAD were only better than DAD. However, whether obvious language disorder exists in this subtype remains to be further confirmed with a larger AD sample size and more persuasive evaluation indexes in the future. 

The disease duration of LTAD was significantly different from those of DAD and OSAD. In the longitudinal analysis, it appeared found that LTAD had a higher rate of change in the thickness of the occipital cortex. Therefore, some LTAD subjects may convert to DAD. As proved by the previous studies, it is in the left hemisphere that the atrophy will take place firstly, followed by the diffusion atrophy throughout the brain during the disease duration [33,34]. This is also consistent with the fact that TAD will account for a large proportion in late AD.

The proportions of APOE ε4 and APOE ε2 carried in MAD were the highest. According to a recent study, it is speculated that APOE ε2 has protective effects on the brain structure and cognition and may retard the cortical atrophy and cognitive degradation from APOE ε4 [35]. However, this conclusion cannot be drawn through only a few studies of atrophy subtypes yet. The proportions of abnormal Aβ_1–42_ and tau carried in OSAD were the highest, and abnormal CSF levels could accelerate the disease process [36]. According to previous studies, the HSAD, which is similar to OSAD, is the subtype with the fastest progression. The atrophy degree of OSAD was not as high as that of DAD, but the CSF levels were the worst. It also proved that our results reflected the subtype differences, but not the difference in the degree of atrophy. No significant difference of tau was found in our study, which coincides with several other studies [5,6]. No difference was found either in this study through the quantitative comparison, but it was found through the qualitative comparison that OSAD was significantly different from MAD, indicating that the CSF levels could be comprehensively investigated by combining the quantitative and qualitative analyses.

The four subtypes showed no significant differences in age or education level. Nevertheless, such differences have been found in some of the previous studies [6,28]. OSAD, which was similar to HSAD, did not differ from other subtypes in age, but in most of the other studies, HSAD was a subtype that younger people are susceptible to [6,15,28], while the opposite result has been obtained in a study [8]. The inconsistent conclusions may be related to whether the covariable is eliminated, or the elimination method of covariant, besides the data and research method. For instance, AD samples have been taken as a whole to eliminate the covariable in some studies [28], while the method similar to that in this study has been used in some other studies [14,29], namely the covariable has been eliminated by using AD to establish a regression model for CN. In addition, the selection of covariable is also varied. For example, only ICV has been used as the covariable in some studies, while gender, age, etc. have been chosen in some other studies. Furthermore, it is difficult to accurately evaluate the influences of covariables on the cerebral structure of AD, and no perfect solution to this problem has been proposed yet.

Various significant differences were reflected among the subtypes in the ADNI composite scores, but significant differences appeared only in few subitems. Therefore, the composite score was more sensitive than the subitems in our subtype results. In our study, the difference was significant in FAQ, which, however, was not included into the ADNI composite score. The non-negligible problem is that both composite score and other cognitive scales (e.g., MMSE) are mainly used to investigate the differences among CN, MCI, and AD [37], but they are not specialized for differentiating AD subtypes. In the future, more factors such as neuropsychology, neuropathology, and brain structure should be combined to construct composite scores more suitable for reflecting the differences of AD subtypes.

According to the baseline data of ADNI, it was found in our study that most AD subjects followed a certain subtype, which would not be changed with the duration of disease, indicating that each subtype defined in our study does not simply refer to a stage of the disease course. However, other subjects would be altered into other subtypes, which might be ascribed to different underlying pathophysiological factors among the subjects, leading to a certain subtype tendency during the data collection. This also coincides with the soft classification of fuzzy clustering in MOE. Given this, the AD heterogeneity can be more comprehensively explored in the future study from the perspective of multi-subtype attribution by mining the potential factors among the subjects.

In the SVM analysis of MOE, the classification accuracy was the highest for DAD and CN with the most serious atrophy, and the lowest for MAD. The specificity of the DAD was very low. A side effect of clustering was that SVM might be confounded with MOE, and, from the angle of classification model, it was not as complicated as the specialized model in the research on AD subtype classification. However, it could be seen from the results of four SVMs, the heterogeneity indeed existed among the AD samples. In most of the research on AD classification and recognition, AD has been regarded as a single pattern. It is anticipated that applying the study results of AD subtypes to AD classification research may provide a clue for improving the precision of AD classification model and enhancing the interpretability, this, however, has not been verified in other studies of AD subtypes.

Some limitations still exist in our study. First, the voxel-by-voxel whole brain voxel-based morphometry (VBM) measurements, which have been widely used in the studies of AD. Both methods provide different types of information and should thus be used in tandem. In future study, the MOE can be used to define AD subtypes based on the features of VBM, which can further compare with the conclusions of this study. Second, more and more studies have shown that the brain diseases of the aged are complicated, and multiple comorbid pathologies are quite common [38,39]. In a recent study, an AD subtype—limbic-predominant age-related TAR DNA-binding protein 43 (TDP-43) encephalopathy (LATE)—has been defined [40]. LATE, which has similar symptoms to AD, is not caused by the accumulation of β-amyloid proteins but by the deposition of TDP-43 protein in brain, and it mainly affects the age group of over 80 years old. The TDP-43 deposition is firstly developed from the amygdala to the hippocampus, which is identical to many conclusions regarding the limbic-predominant in the studies of AD subtype [4]. In our subtype defined results, the proportion of over 80-year-old subjects with LTAD, which was the most approximate to limbic-predominant AD, was 36.0% (OSAD: 28.60%, MAD: 25%, DAD: 30.60%). It is speculated that LATE and AD may co-exist in some subjects in LTAD or limbic-predominant AD. However, as our study data were at early stage of AD and the number of LTAD subjects was limited, it was difficult to obtain a determinate conclusion yet. Nevertheless, this provides a new idea to study the AD subtypes in the future. The subjects at late stage of AD or autopsy specimens can be included in the future study of AD subtype, and then our conclusions can be combined to further figure out how various factors are inducing AD start interacting with each other and co-occurring, which will facilitate the better prevention, diagnosis, and prevention of diseases.

## 5. Conclusions

The atrophy patterns of gray matter from sMRI were input into MOE to simulate the heterogeneous cerebral cortex change process from CN to AD. Four AD subtypes were identified, and their structural, neuropathological, and neuropsychological characteristics were analyzed. Subsequently, the differences of each AD subtype and CN linear classifier were compared and interpreted in the heterogeneous models. The results of our study show that substantial heterogeneity exists among the AD subjects. An asymmetrical atrophy pattern was discovered in the AD cohorts, the extreme proportions of APOE were carried, and the highest CSF characteristics were manifested in OSAD. It has been discovered through the cognitive assessment that the composite scores may be of high sensitivity for the AD subtypes. The differences between the conversion models can be obtained in the aspect of the atrophy pattern, thus better expounding the potential physiological processes in the development of AD and improving the personalized diagnosis.

## Figures and Tables

**Table 1:** Demographic, vitals, and cognitive characteristics of the study subtypes.

Characteristics	CN	AD	OSAD	LTAD	MAD	DAD	*p*-Values
*n* (%)	228	192	56 (29.2%)	43 (22.4%)	31 (16.1%)	62 (32.3%)	
Age (years)	75.9 ± 5.0	75.4 ± 7.4	75.0 ± 7.8	76.3 ± 7.1	74.4 ± 7.7	75.6 ± 7.3	0.720
Women, *n* (%)	109 (47.8%)	91 (47.4%)	28 (50.0%)	13 (30.2%)	15 (48.4%)	35 (56.5%)	0.029 ^a,e,g^
Education (years)	16.1 ± 2.9	14.7 ± 3.1	14.7 ± 3.5	15.1 ± 2.6	14.3 ± 7.7	14.6 ± 3.0	0.680
Age of onset (years)		70.0 ± 14.5	68.5 ± 16.3	70.0 ± 18.0	71.7 ± 7.9	70.6 ± 12.5	0.524
Disease duration (years)		3.1 ± 2.7	3.5 ± 2.9	2.3 ± 2.2	2.7 ± 2.2	3.6 ± 2.8	0.032 ^a,e^
Left-handedness	17 (7.5%)	11 (5.7%)	5 (8.9%)	3 (7.0%)	0	3 (4.8)	0.342 ^g^
BMI	26.7 ± 4.3	25.5 ± 3.9	26.1 ± 4.4	25.3 ± 3.8	25.1 ± 3.3	25.3 ± 3.7	0.561
Systolic (mmHg)	134.5 ± 16.9	137.6 ± 17.1	137.7 ± 16.2	140.3 ± 18.6	139.7 ± 19.0	137.7 ± 16.2	0.294
Diastolic (mmHg)	74.6 ± 10.3	73.8 ± 9.9	72.9 ± 8.7	73.3 ± 11.5	74.1 ± 9.7	74.7 ± 9.9	0.774
Pulse rate (per minute)	67.0 ± 10.8	63.7 ± 9.0	65.0 ± 7.9	62.3 ± 8.1	61.4 ± 10.8	64.5 ± 9.3	0.461
Respirations (per minute)	16.8 ± 3.2	17.0 ± 3.1	17.2 ± 2.0	17.1 ± 3.5	17.4 ± 2.9	16.5 ± 3.0	0.181
MMSE	29.1 ± 1.0	23.3 ± 2.0	23.9 ± 1.9	24.0 ± 2.0	24.0 ± 1.8	22.6 ± 2.1	<0.001 ^e,f^
CDR-SB	0.03 ± 0.12	4.3 ± 1.6	4.2 ± 1.5	4.0 ± 1.4	3.8 ± 1.5	4.8 ± 1.8	0.011 ^e,f^
FAQ	0.14 ± 0.6	12.9 ± 6.9	12.6 ± 6.3	11.9 ± 6.4	8.9 ± 5.6	15.9 ± 7.1	<0.001 ^b,c,d,e,f^
ADAS-Cog 11	6.2 ± 2.9	18.5 ± 6.3	18.2 ± 6.7	17.5 ± 6.0	15.6 ± 4.0	21.0 ± 6.3	<0.001 ^c,e,f^
ADAS-Cog 13	9.5 ± 4.2	28.8 ± 7.6	28.7 ± 7.9	27.0 ± 7.1	25.4 ± 5.7	31.8 ± 7.6	<0.001 ^c,e,f^
ADNI-MEM	0.97 ± 0.53	−0.84 ± 0.55	−0.89 ± 0.54	−0.69 ± 0.45	−0.60 ± 0.57	−1.03 ± 0.56	<0.001 ^e,c,b,f^
ADNI-EF	0.64 ± 0.75	−0.96 ± 0.89	−0.91 ± 0.78	−0.85 ± 0.98	−0.80 ± 0.94	−1.13 ± 0.91	0.293
ADNI-LAN	0.78 ± 0.75	−0.78 ± 0.89	−0.67 ± 0.90	−0.65 ± 0.88	−0.51 ± 0.71	−1.10 ± 0.90	0.05 ^c,e,f^
ADNI-VS	0.23 ± 0.60	−0.60 ± 0.91	−0.55 ± 0.80	−0.52 ± 0.79	−0.57 ± 0.96	−0.72 ± 1.04	0.645

BMI, Body Mass Index. ^a:^ significant differences (*p* < 0.05) between OSAD and LTAD; ^b:^ significant differences (*p* < 0.05) between OSAD and MAD; ^c:^ significant differences (*p* < 0.05) between OSAD and DAD; ^d:^ significant differences (*p* < 0.05) between LTAD and MAD; ^e:^ significant differences (*p* < 0.05) between LTAD and DAD; ^f:^ significant differences (*p* < 0.05) between MAD and DAD; ^g:^ The $\chi^{2}$ test was used.

**Table 2:** Neuropathological characteristics of the study subtypes.

Characteristics	CN	AD	OSAD	LTAD	MAD	DAD	*p*-Values
APOE ε4 (n(carry%))	60 (26.3%)	127 (66.1%)	38 (67.9%)	27 (62.8%)	22 (71.0%)	40 (64.5%)	0.962 ^f^
1	55 (24.1%)	91 (47.4%)	29 (51.8%)	19 (44.2%)	16 (51.6%)	27 (43.5%)	
2	5 (2.1%)	36 (18.8%)	9 (16.1%)	8 (18.6%)	6 (19.4%)	13 (21.0%)	
APOE ε2 (n(carry%))	21 (9.2%)	14 (7.3%)	3 (5.4%)	1 (2.3%)	7 (22.6%)	3 (4.8%)	<0.018 ^b,c,e,f^
Aβ_1–42_ (ng/L)	205.8 ± 54.7	143.6 ± 40.6	132.0 ± 25.5	150.6 ± 41.7	168.5 ± 55.6	139.7 ± 41.4	0.033 ^b,e^
Aβ_1–42_ (abnormal%)	44 (37.6%)	89 (91.3%)	31 (97.0%)	21 (84.0%)	10 (76.9%)	26 (93.9%)	0.151 ^f^
n missing	111 (48.7%)	94 (49.0%)	24 (42.9%)	18 (41.9%)	18 (58.0%)	34 (54.8%)	
t-tau (ng/L)	69.7 ± 29.8	121.4 ± 57.6	131.9 ± 54.8	129.4 ± 69.5	88.1 ± 51	118.4 ± 49.3	0.112
t-tau (abnormal%)	21 (17.6%)	64 (63.3%)	25 (78.1%)	17 (68.0%)	4 (30.8%)	18 (54.5%)	0.014 ^b,f^
n missing	109 (47.8%)	96 (50.0%)	24 (42.9%)	19 (44.2%)	18 (58.0%)	35 (56.5%)	
p-tau (ng/L)	25.1 ± 14.6	41.5 ± 19.9	41.53 ± 17.8	41.88 ± 19.4	31.8 ± 19.2	44.8 ± 21.6	0.254
p-tau (abnormal%)	42 (35.3%)	90 (87.4%)	30 (93.8%)	23 (92.0%)	8 (61.5%)	30 (90.9%)	0.015 ^b,f^
n missing	94 (41.2%)	89 (46.4%)	24 (42.9%)	18 (41.9%)	18 (58.0%)	29 (46.8%)	

^a:^ significant differences (*p* < 0.05) between OSAD and LTAD; ^b:^ significant differences (*p* < 0.05) between OSAD and MAD; ^c:^ significant differences (*p* < 0.05) between LTAD and MAD; ^d:^ significant differences (*p* < 0.05) between LTAD and DAD; ^e:^ significant differences (*p* < 0.05) between MAD and DAD; ^f:^ The $\chi^{2}$ test was used.

## Data Availability

Not applicable.
